# Examining the relationship between social determinants of health, measures of structural racism and county-level overdose deaths from 2017–2020

**DOI:** 10.1371/journal.pone.0304256

**Published:** 2024-05-23

**Authors:** Zoe Lindenfeld, Diana Silver, José A. Pagán, Donglan Stacy Zhang, Ji Eun Chang

**Affiliations:** 1 Department of Public Health Policy and Management, School of Global Public Health, New York University, New York, NY, United States of America; 2 Division of Health Services Research, New York University Long Island School of Medicine, Mineola, New York, United States of America; Weill Cornell Medicine, UNITED STATES

## Abstract

**Introduction:**

Despite being an important determinant of health outcomes, measures of structural racism are lacking in studies examining the relationship between the social determinants of health (SDOH) and overdose deaths. The aim of this study is to examine the association between per capita revenue generated from fines and forfeitures, a novel measure of structural racism, and other SDOH with county-level overdose deaths from 2017–2020.

**Methods:**

This longitudinal analysis of 2,846 counties from 2017–2020 used bivariate and multivariate Generalized Estimating Equations models to estimate associations between county overdose mortality rates and SDOH characteristics, including the fines and forfeitures measure.

**Results:**

In our multivariate model, higher per capita fine and forfeiture revenue (5.76; CI: 4.76, 6.78), households receiving food stamps (1.15; CI: 0.77, 1.53), residents that are veterans (1.07; CI: 0.52, 1.63), substance use treatment availability (4.69; CI: 3.03, 6.33) and lower population density (-0.002; CI: -0.004, -0.001) and percent of Black residents (-0.7`; CI: -1.01, -0.42) were significantly associated with higher overdose death rates. There was a significant additive interaction between the fines and forfeitures measure (0.10; CI: 0.03, 0.17) and the percent of Black residents.

**Conclusions:**

Our findings suggest that structural racism, along with other SDOH, is associated with overdose deaths. Future research should focus on connecting individual-level data on fines and forfeitures to overdose deaths and other health outcomes, include measures of justice-related fines, such as court fees, and assess whether interventions aimed at increasing economic vitality in disadvantaged communities impact overdose deaths in a meaningful way.

## Introduction

The national overdose crisis continues to devastate communities across the United States (US). In 2021, there were over 107,000 deaths due to drug overdose reported in the US, an increase of more than 10,000 deaths from the previous year [1]. The overdose epidemic has been conceptualized as a community-level crisis, particularly when contextualized in the wider disenfranchisement of the American working class and other “deaths of despair” (i.e., deaths due to suicide, overdose, or alcoholic liver disease) in communities with weakened economies) [2]. In looking for ways to reduce substance misuse and its consequences, scholars and policymakers have focused attention on the social determinants of health (SDOH), or the non-medical factors that affect health outcomes [3, 4]. SDOH includes factors such as housing instability, the neighborhood and built environment, food insecurity, and access to transportation [3, 4], as well as structural racism, which works to negatively impact population health outcomes through reinforcing discriminatory beliefs, laws, and decisions related to resource allocation, including access to high-quality health care [5].

Recent studies have evaluated the role of community-level SDOH in the risks associated with substance use and substance use disorders (SUDs). These studies have found that overdose deaths are higher in counties with greater income inequality [6–9] and violent crime rates [7], as well as counties with lower access to the Internet and access to health care [7]. However, community-level measures of structural racism have not been well-explored in studies of overdose deaths, and sound measures of structural racism are lacking in public health research overall. For example, studies using measures of segregation in assessing health outcomes have found a detrimental impact of racism on population health [5, 10], though these studies note that racial segregation reflects the composition of the community, rather than the structural factors influencing that context [10].

Previous research has found that fines and forfeitures (obtained from, for example, violations of the law, civil penalties, and forfeited bail/collateral), which are instituted in counties that have the judicial and police capacity to carry out their collection, disproportionately affect marginalized populations, and can be operationalized as a measure of structural racism. In part, as documented in a report written by the States Department of Justice following the murder of Michael Brown Jr. in Ferguson [11], this is because Black and minority residents have historically been targeted for low-offense citations that generate fines [12–14]. Indeed, studies have found that a city’s reliance on fines and forfeitures as a source of revenue is strongly related to the percent of residents who are Black [14], as well as the overrepresentation of White officers in local police departments [15]. Additionally, studies have found that having a higher percentage of revenue generated from fines and forfeitures impacts financial stability [16], homelessness [17], and criminal justice involvement [18] among community residents. These outcomes have also been associated with heightened overdose mortality risk, with studies finding that individuals in poverty, experiencing homelessness or housing insecurity, and with prior criminal justice involvement are at the highest risk for experiencing a fatal overdose [19–21]. Some scholars estimate that billions of dollars flow from vulnerable communities to governments each year through this mechanism [16]. However, only a few studies have examined the impact of this practice on health outcomes [12].

Here, we examine the association of structural racism and other SDOH with county-level overdose deaths from 2017–2020 by including a measure of the revenue generated by fines and forfeitures per capita. This study demonstrates how fines and forfeiture revenue can be used within health disparities research as a novel measure of structural racism, and provides insight into how having a higher reliance on revenue generated from monetary fines can disproportionately target and impact the health of vulnerable communities.

## Methods

### Data sources

#### County-level measure of structural racism

We obtained measures of revenue generated from fines and forfeitures from the US Census Bureau’s Census of Governments, which collects information on state and local governments’ expenditures and revenues [22]. A survey of the full sample of all county and sub-county governments is conducted every five years, while a limited sample is conducted annually. As part of this survey, the census asks governments how much revenue is generated from fines and forfeitures, defined as “penalties imposed for violation of law; civil penalties (e.g., for violating court orders); court fees if levied upon conviction of a crime or violation… and forfeits of deposits held for performance guarantees or against loss or damage (such as forfeited bail and collateral).”[22] We obtained this measure for all county and subcounty governments from 2017–2020, which we converted to a per capita measure using population estimates from the American Community Survey (ACS) [23]. Following prior research, we assigned city and county municipal governments to counties and excluded municipalities without expenditures associated with police or judicial operations, which are necessary for the collection of fines and forfeitures [14]. We also excluded counties with less than 2,500 inhabitants [14]. To account for the skewed distribution of the raw measure of fine and forfeiture revenues, we took the logarithm of this measure plus one [12, 14].

#### Overdose mortality

Data on drug overdoses were obtained from the Centers for Disease Control and Prevention (CDC) Restricted Mortality Files [24]. Mortality data collected by the CDC are based on death certificates for US residents which are coded by states and sent to the CDC National Center for Health Statistics. Each death certificate identifies underlying causes of death using the International Classification of Diseases, Tenth Revision (ICD-10) codes [25]. Analyses are restricted to deaths with ICD–10 codes for drug overdoses: X40–X44, X60–X64, X85, and Y10–Y14. Observations were aggregated at the county level for each year of data (2017–2020) and converted to a death rate per 100,000 population using county-level population estimates from the ACS [23].

#### County SDOH characteristics

Data on county-level SDOH were obtained from the Agency for Healthcare Research and Quality (AHRQ) SDOH database [26]. The AHRQ database uses a five-domain framework to categorize SDOH variables at the community-level: 1) social context; 2) economic context; 3) education; 4) physical infrastructure; and 5) healthcare context [26]. SDOH within these domains include demographic variables, income, employment, educational achievement, literacy, housing, transportation, and insurance coverage. Variables are drawn from 44 sources, including the ACS, the Food Environment Atlas, CDC Places, Provider of Services, and County Health Rankings. There are over 17,000 variables available in the AHRQ database across years at the county-level. We included variables that were available each year from 2017–2020 and had a missingness <60%, and excluded variables capturing the same concept as included variables (e.g., categorical age and continuous age, separate values for male and female). This process resulted in the inclusion of 78 variables from the AHRQ database in our final dataset. We also calculated a county-level measure of population density using land area estimates from the US Census Bureau and population estimates from the AHRQ database.

#### Statistical analysis

We reported descriptive statistics capturing the county-level mean for the overdose rate outcome variable, the per capita fine and forfeiture measure, and each SDOH variable for each year of data. Bivariate analyses estimate the relationship between county-level overdose deaths, county level per capita fines and forfeitures, and each SDOH variable.

Using the most complete year of data (2017) we conducted least absolute shrinkage and selection operator (LASSO) on a model with 19 predictors representing the five domains of the AHRQ framework for variable selection, which resulted in the inclusion of 13 variables in our final model. We used generalized estimating equations (GEE) for longitudinal data to estimate associations between county SDOH characteristics, which included county-level per capita revenue generated from fines and forfeitures longitudinally, as well as an interaction term of the percent of Black residents in a county and the per capita fine and forfeiture revenue, with county-level overdose mortality rate per 100,000. GEE is an extension of generalized linear models to longitudinal data that takes into account correlation within observations (counties) [27]. We then calculated the average marginal difference in predicted per capita fine and forfeiture revenue holding the percent of Black residents in a county at the mean, the mean plus one standard deviation, and the mean minus one standard deviation. Statistical analyses were performed in Stata SE 17 with statistical significance determined at p < .05 [28]. This study was deemed exempt from Institutional Board Review at New York University.

## Results

**Fig 1:**
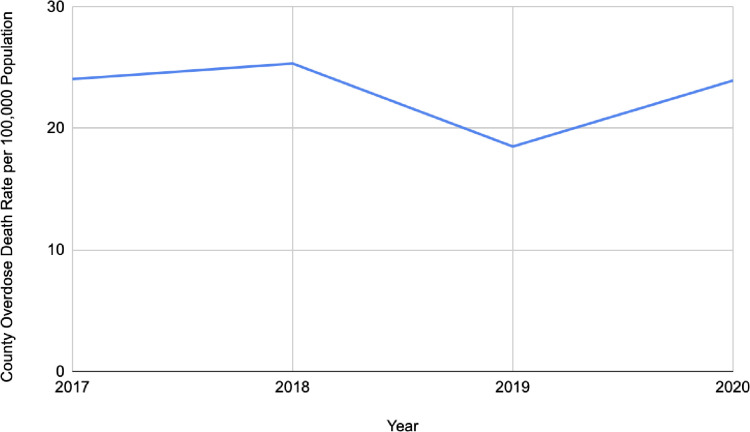
County-level overdose death rates over time from 2017–2020 among counties included in our sample*. * There were n = 2,795 counties included in 2017, n = 2,046 in 2018, n = 2,029 in 2019, and n = 2,031 in 2020.

**Fig 2:**
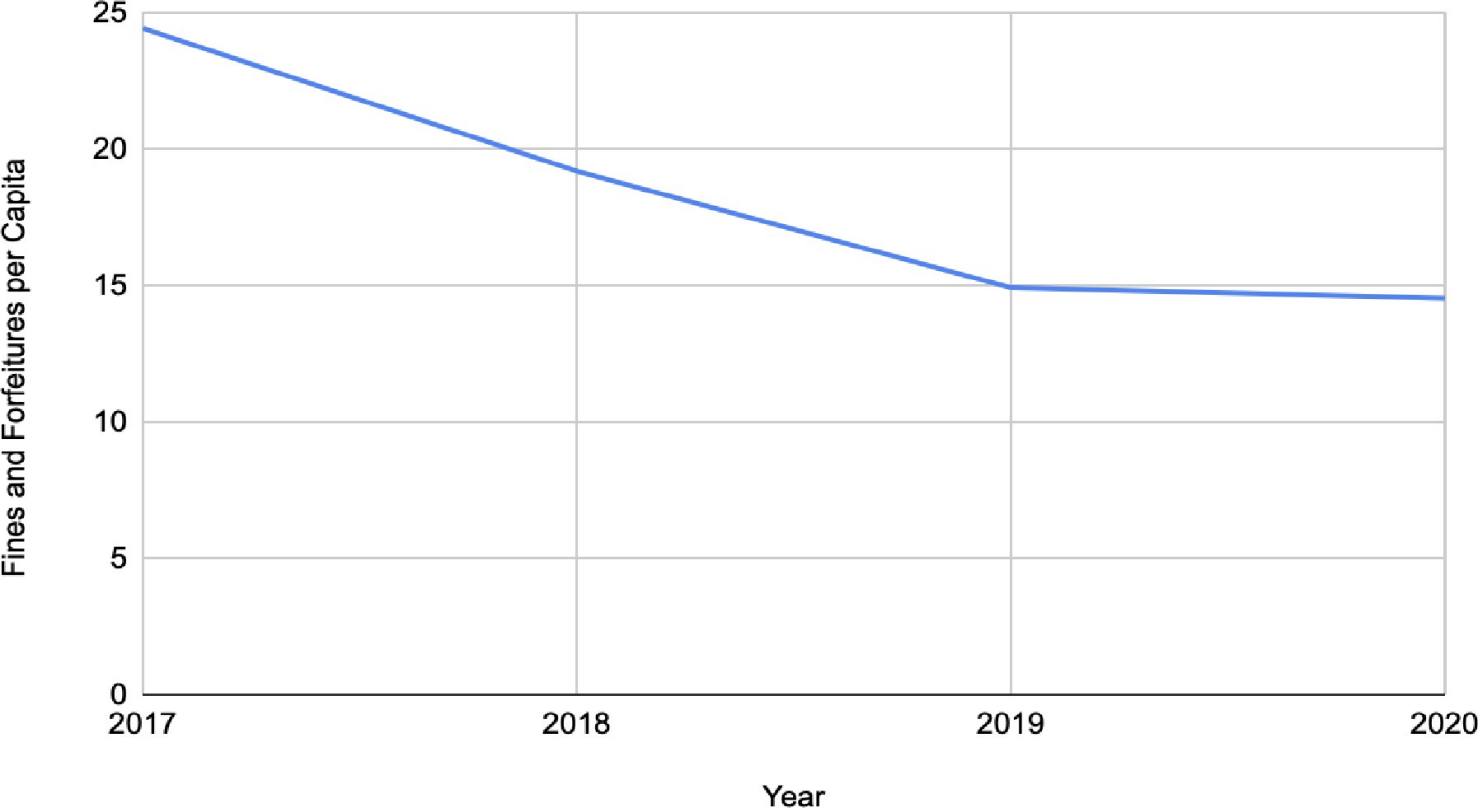
County-level per capita fine and forfeiture revenue over time from 2017–2020 among counties included in our sample. * There were n = 2,795 counties included in 2017, n = 2,046 in 2018, n = 2,030 in 2019, and n = 2,031 in 2020.

The study sample included 2,796 counties in 2017, 2047 in 2018, 2031 in 2019, and 2033 in 2020, summing to 8,900 observations in the full longitudinal sample with representation from all 50 US states. Across all years, the mean county-level overdose death rate among counties included in our sample was 23.06 deaths per 100,000 persons. Fig 1 shows the mean county-level overdose death rate each year, which was 24.05 deaths per 100,00 persons in 2017, 25.33 in 2018, 18.5 in 2019, and 23.93 in 2020. Fig 2 shows the variation in per capita revenue generated from fines and forfeitures over time, which was 24.31 in 2017, 19.18 in 2018, 14.91 in 2019, and 14.52 in 2020. As indicated in Table 1, across all years, the mean amount of fines and forfeitures per capita was $18.79 (SD: 47.73), the mean percentage of Black residents was 9.75 (SD: 14.67), uninsured residents 9.76 (SD: 4.76), residents with poverty status determined 14.36 (SD: 5.87), households receiving food stamps 13.30 (SD: 6.13), Hispanic/Latinx residents 9.13 (12.89), residents that were veterans 9.79 (SD: 2.67) residents with a Bachelor’s degree (ages 25 and over) 14.88 (SD: 5.86), residents with less than a high school diploma (ages 25 and over) 12.71 (5.90) and percent of housing units that were mobile homes was 11.81 (SD: 9.26). The majority of counties in our sample were located in the South of the U.S (44.99%), with lower percentages in the Northeast (8.00%), Midwest (31.10%), and West (15.91%). The mean population density was 129.96 residents per square mile of county land area, and the average amount of SUD treatment facilities with three medications for opioid use disorders (MOUD) available was 0.19 (SD: 0.95).

**Table 1:** Descriptive statistics and results from adjusted and unadjusted longitudinal GEE models predicting overdose death rates from 2017–2020.

	Descriptive Statistics (mean and SD)	Unadjusted GEE (95% CI) (n = 8,900 observations, n = 2,846 groups)↟	Adjusted GEE (95% CI) (n = 8,900 observations, n = 2,846 groups)↟
Fine and forfeitures per capita☨	18.79 (47.73)	5.64 4.80, 6.48)**	5.76 (4.76,6.78)**
Percent Black	9.75 (14.67)	0.06 (-0.02, 0.14)	-0.71 (-1.01, -0.42)
Percent Hispanic/Latinx	9.13 (12.89)	-0.09 (-0.18, 0.01)	-0.18 (-0.32, -0.05)**
Percent veterans	8.79 (2.67)	0.799 (0.32, 1.27)**	1.07 (0.52, 1.63)**
Percent with poverty status determined	14.36 (5.87)	0.20 (-0.01, 0.42)	0.39 (-0.02, 0.82)
Percent of households receiving food stamps	13.30 (6.13)	0.50 (0.30, 0.71)**	1.15 (0.77, 1.53)**
Percent with Bachelor’s degree (> = 25 years of age)	14.88 (5.86)	0.27 (0.06, 0.48)*	0.14 (-0.23, 0.52)
Percent with less than a high school diploma (> = 25 years of age)	12.71 (5.90)	-0.28 (-0.49, -0.06)**	-0.11 (-0.59, 0.37)
Population density	129.96 (739.27)	0.001 (0-.0004, 0.002)	-0.002 (-0.004, -0.001)**
Region (%) Northeast South Midwest West	8.00 (712) 44.99 (4,005) 31.10 (2,768) 15.91 (1,416)	*Ref* -2.19 (-7.02, 2.63) -6.50 (-11.49, -1.51)* -1.51 (-6.97, 3.93)	*Ref* 6.29 (0.77, 11.80)* -2.90 (-7.99, 2.18) 0.48 (-5.36, 6.34)
Percent of households living in mobile homes	11.81 (9.26)	-0.37 (-0.50, -0.23)**	-0.70 (-0.90, -0.51)**
Percent uninsured	9.76 (4.76)	-0.54 (-0.80, -0.27)**	-0.86 (-1.21, -0.52)**
Number of SUD treatment facilities offering three MOUDs	0.19 (0.85)	5.29 (3.82, 6.76)**	4.69 (3.03, 6.33)**
Interaction: per capita fine and forfeiture and percent Black	—	—	0.10 (0.03, 0.17)**

*p<0.05

**p<0.01

☨Log transformed + 1 in unadjusted and adjusted models

**↟**Average of 3.6 observations per group, min = 1 and max = 4

S1 Fig. displays the variables organized by domain and our variable selection process. In unadjusted GEE models, fines and forfeitures per capita (5.64; CI: 4.80, 6.48), percent of households receiving food stamps (0.50; CI: 0.30, 0.71), percent of individuals aged 25 and older with a Bachelor’s degree (0.27; CI: 0.06, 0.48), percent of residents that are veterans (0.79; CI: 0.32, 1.27) and the amount of SUD treatment facilities with three MOUDs (4.29; CI: 3.82, 6.76) were positively and significantly associated with the county-level overdose death rate. Percent uninsured (-0.54; CI: -0.80, -0.27), percent of households living in mobile homes (-0.37; CI: -0.50, -0.23), percent of residents aged 25 and over with less than a high school diploma (-0.28; CI: -0.49, -0.06) and being located in the Midwest compared with the Northeast (-6.50; CI: -11.49, -1.51) were negatively and significantly associated with the county-level overdose death rate.

In the fully adjusted GEE model, higher county-level overdose death rates were significantly associated with higher per capita fines and forfeiture revenue (5.76; CI: 4.76, 6.78), percent of households receiving food stamps (1.15; CI: 0.77, 1.54), the percent of residents that were veterans (1.07; CI: 0.52, 1.63), being located in the South compared to the Northeast (6.29; CI: 0.77 11.88), and the amount of SUD treatment facilities with three MOUDs (4.69; CI: 3.03, 6.33). Having a lower percent of Black residents (-0.71; CI: -1.01, -0.42), Hispanic/Latinx residents (-0.18; CI: -0.32, -0.05) uninsured residents (-0.86; CI: -1.21, -0.52), housing units that are mobile homes (-0.70; CI: -0.90, -0.50), as well as lower population density (-0.002; CI: -0.004, -0.001), were significantly associated with having a higher overdose rate on the county level. There was a significant additive interaction between the percent of Black residents in a county and the amount of per capita revenue generated from fines and forfeitures (0.10; CI: 0.03, 0.17). The marginal estimates reveal predicted per capita fine and forfeiture revenue was higher when the percent of Black residents was held at one SD above the mean for all counties, and lower when the percent of Black residents was held at one SD below the mean for all counties (see S2 Fig).

## Discussion

This study evaluated the impact of SDOH, including a novel measure of structural racism, on a county level on drug overdose mortality over a four-year period. Consistent with prior studies, we found that counties with a lower population density and percentage of residents that are racial/ethnic minorities had higher rates of deaths due to drug overdose [29]. We also found that higher county-level overdose death rates were associated with a higher number of SUD treatment facilities offering MOUD, having a higher percentage of households receiving food stamps, and notably, having a higher amount of per capita revenue generated from fines and forfeitures, controlling for other community level factors.

The finding that higher per capita revenue from fines and forfeitures was associated with a higher county overdose death rate can be understood in the context of a growing literature documenting the detrimental effects of fines and forfeitures on vulnerable communities. Because the majority of financial sanctions are concentrated among low-income populations facing financial difficulties, several scholars have noted that fines and forfeits operate as ‘poverty traps’ by further burdening individuals in poverty with debt accrual [12, 13, 16]. Consequences for being unable to pay include incarceration, having a license revoked, losing the ability to vote, and being ineligible for government jobs [13, 30]. Aside from further entrapping individuals in poverty, these penalties affect a person’s ability to work, gather socioeconomic resources, and attend health appointments, and add distress to their life [12]. These factors, particularly incarceration [21, 31], poverty [19], and disrupted access to health care [21], have been associated with heightened risk for overdose death at the individual level, with studies finding that rates of overdose mortality and substance misuse are higher in economically depressed areas [32–34].

Notably, while the percentage of Black residents in a county was negatively associated with overdose deaths, this relationship was moderated by having higher per capita revenue generated from fines and forfeitures. Because the opioid epidemic began with the overprescribing and overmarketing of prescription opioids in states with large White populations such as West Virginia, Maine, and Kentucky [35], the epidemic has been conceptualized as a problem affecting poor, rural, White communities. For example, the aggressive marketing tactics used by pharmaceutical companies to promote opioids to physicians in the late 1990s and early 2000s led to substantial increases in opioid prescription patterns in these states, with opioids such as oxycodone prescribed up to five more than the national average [35]. The notion that the overdose epidemic is concentrated in rural, White communities is substantiated by our finding of population density being significantly negatively associated with overdose deaths, and the percent of households receiving food stamps being positively associated with overdose deaths. However, evidence also suggests that the impact of the opioid epidemic on Black Americans have been underreported, with the recent increase in overdose deaths due to heroin or fentanyl disproportionately affecting Black communities [36]. Our finding that overdose rates are significantly higher in counties with both a higher percentage of Black residents and per capita fine and forfeiture revenue suggests that structural forces, including racialized policing and enforcement of monetary sanctions, may be contributing to disparities in overdose deaths in communities with a higher percentage of Black residents, along with other factors that impact detrimental substance use outcomes in Black communities, including a lack of funding and resources for SUD treatment and other social services [37].

Our study has several limitations. First, because the Census of Governments data used in our analysis includes years in which there was a limited census conducted (2018–2020), the counties included may be subject to selection bias; however, we do include on year of data (2017) in which a full survey was conducted, which is a strength to this analysis. Second, while the GEE model accounts for time and intra-unit correlation effects, we do not include data on the implementation of state policies (i.e., Medicaid 1115 waivers for substance use disorders or Good Samaritan laws) [38] which may affect overdose risk in communities. Third, the fines and forfeitures data taken from the Census of Governments do not include other court costs, such as fees for jail time, and is likely an underestimation of the effect. Similarly, while the Census of Governments is a reputable survey, the accuracy of the data relies on the accuracy of the governments reporting their revenues, which may be subject to reporting bias and bias our results towards the null. Additionally, because our analysis was at the county level, it is subject to ecological fallacy, and associations may not translate to inferences regarding individual-level risk. Furthermore, while we utilize the amount of revenue generated from fines and forfeiture to measure an important construct of structural racism, this measure cannot fully capture the complexity of structural racism, which is multifaceted in nature [10]. Finally, while our study demonstrates important associations regarding the amount of revenue generated from fines and forfeitures and overdose deaths, our methods do not establish causal relationships, and it is possible that additional factors may be influencing the outcome in our study.

Despite these limitations, this study provides novel insights into socioeconomic and structural forces at the community (county) level that are associated with higher overdose death rates. In recent years, there have been growing attention from federal, state, and local governments aimed at reducing the detrimental impacts of the opioid epidemic [39, 40]. Our finding that the number of SUD treatment facilities are higher in counties with a higher overdose rate is evidence of a response to need and adoption of interventions which increase access to evidence-based SUD treatment. However, our findings suggest that a wider, non-medical, response to the opioid epidemic is necessary.

## Conclusions

Our findings suggest that structural racism, measured by the amount of revenue generated from fines and forfeitures, along with other social determinants of health, is associated with overdose deaths in counties nationwide. Studies that include measures of structural racism which capture policies and practices that disproportionately impact minority groups, such as the amount of revenue generated from fines and forfeiture, alongside other SDOH are needed to assess its impact on community-level health outcomes. Furthermore, there is a need for a broader understanding of how municipal and state strategies that employ monetary sanctions as a form of revenue generation, including funding criminal justice, may disproportionately impact poor and minoritized communities and widen disparities in overdose outcomes. Future research should focus on connecting individual-level data on fines and forfeitures to overdose deaths and other health outcomes, include measures of justice-related fines, such as court fees, and assess whether interventions aimed at increasing economic vitality in disadvantaged communities impact overdose deaths in a meaningful way.

## Supporting information

S1 Fig Variable selection process for SDOH variables included in the final model. (DOCX)

S2 Fig Predicted per capita revenue generated from fine and forfeitures, based on marginal predictions from the longitudinal GEE model holding percent Black residents at the mean, one SD above the mean, and one SD below the mean, 2017–2020 (N  =  8,901). Error bars represent 95% Cis.
(DOCX)
