# Supplementary figures and images for: Examining the relationship between social determinants of health, measures of structural racism and county-level overdose deaths from 2017–2020
Source: PLoS One. 2024 May 23;19(5):e0304256. doi: 10.1371/journal.pone.0304256 (PMC11115243; doi:10.1371/journal.pone.0304256)

**
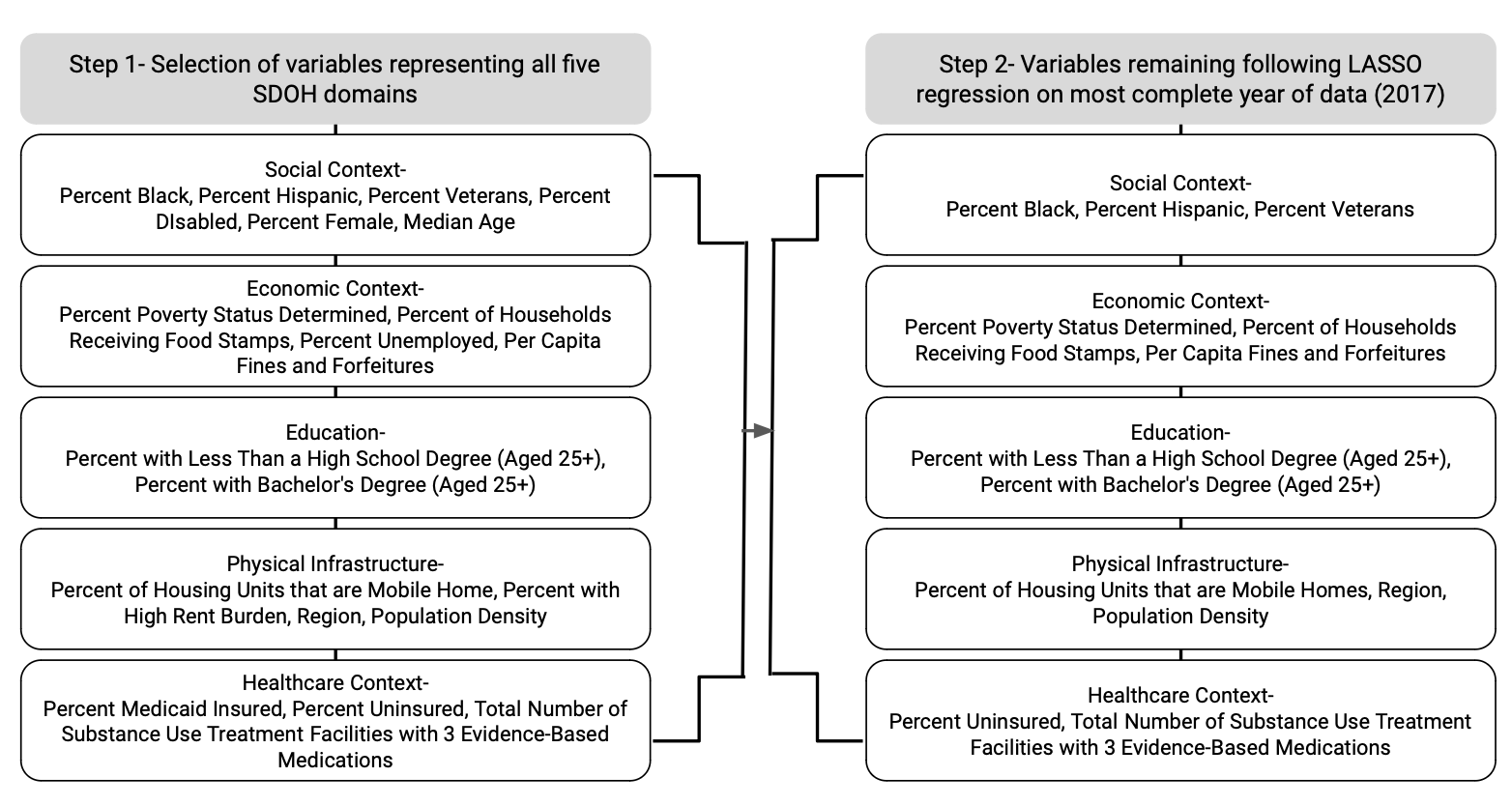
**

Supplement: S1 Fig — (DOCX) [file pone.0304256.s001.docx]

**
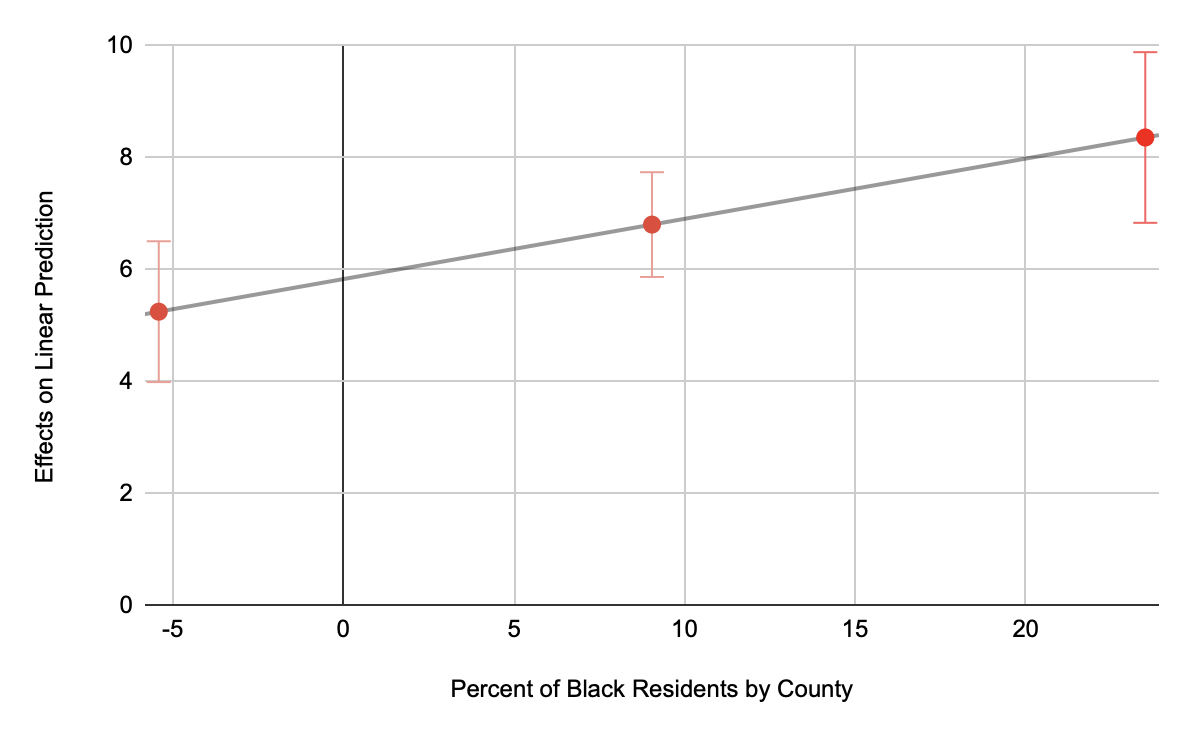
**

Supplement: S2 Fig — Error bars represent 95% Cis. (DOCX) [file pone.0304256.s002.docx]
